# A Prospective Study Comparing Two-Time Points of Thyroid Hormone Replacement during the Holy Month of Ramadan

**DOI:** 10.1155/2019/9843961

**Published:** 2019-07-22

**Authors:** Zeinab Dabbous, Buthaina Alowainati, Sara Darwish, Hamda Ali, Seleena Farook, Mariam Al Malaheem, Abeir Abdalrubb, Wajiha Gul, Wajiha Abu Haliqa

**Affiliations:** Department of Endocrine, Qatar Metabolic Institute, Hamad Medical Corporation, Doha, Qatar

## Abstract

**Background:**

Muslims all over the world fast during the month of Ramadan from dawn until dusk. There is little data regarding the best timing of levothyroxine intake during the month of Ramadan where taking it on an empty stomach represents a challenge to most patients. Our study aims to compare two-time points of levothyroxine intake during Ramadan in terms of change in thyroid stimulating hormone (TSH), compliance, and convenience.

**Study Design and Methods:**

This was an open-label, randomized, prospective trial. Adult patients known to have primary hypothyroidism with stable TSH for the last 6 months who intended to fast during the month of Ramadan were invited to participate in this prospective study. The study took place during Ramadan of H1438 (May-June 2017). All patients were randomly assigned to two groups. In group A (n= 50) patients took levothyroxine 30 minutes before breaking the fast at sunset (iftar), and in group B (n= 46) patients took it 30 minutes before an early morning meal before sunrise (suhour).

**Results:**

TSH levels increased in both group A (from 1.99 to 3.28 mIU/L) and group B (from 1.54 to 3.28 mIU/L) after Ramadan fasting. There was no difference between the two groups. Compliance with intake instructions, all of the time, was reported in 41.6% of group A and 35.7% of group B patients. In both the groups, 95% of patients said it was convenient for them to take the medication at the assigned time.

**Conclusion:**

Choosing an optimal time for levothyroxine intake during the month of Ramadan remains a challenge. The current study did not provide any evidence on ideal time and dose of levothyroxine administration during fasting to manage hypothyroidism. Studies with a larger number of patients need to be done to further explore this issue.

## 1. Introduction

Ramadan is the ninth month of the Islamic calendar, and Ramadan fasting is observed by millions of Muslims all over the world. Ramadan fasting will be 29 or 30 days based on the Islamic lunar calendar [1]. In Ramadan to abstain from food and drinks from dawn (suhour) to sunset (iftar) is considered as a sign of restraint and introspection.

Levothyroxine is generally recommended to be taken on an empty stomach, half an hour before food to prevent interference with its intestinal uptake by either food or medications [2, 3]. The absorption of thyroxine declines from 80% in the fasting condition to 60% in the fed condition [3]. The standard dosage of levothyroxine is 1.5 *μ*g/kg body weight to be taken orally in the fasting state 30 minutes before food [4].

It is difficult to orchestrate such coordination of intake of food after levothyroxine especially during the month of Ramadan. A recent prospective cohort study of 64 hypothyroid patients who observed fasting during the month of Ramadan showed a significant increase in TSH after Ramadan. The change was not affected by the timing of levothyroxine intake [5].

During Ramadan our usual practice is to advice patients to take levothyroxine at iftar time with some water and wait 30 minutes before eating their meal. This has been particularly challenging in recent years where fasting during Ramadan can be up to 15 hours. To date, there is lack of sufficient evidence to guide us on the best timing of levothyroxine intake during Ramadan. We aimed to compare two-time points of levothyroxine intake during Ramadan focusing on change in TSH, compliance, and convenience.

## 2. Patients and Methods

### 2.1. Study Design

**Figure 1:**
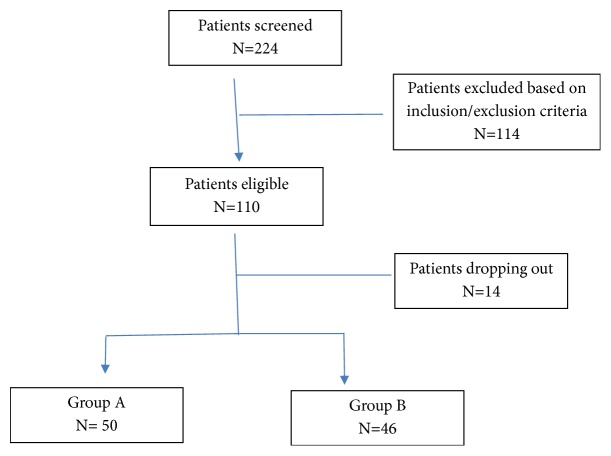
The flowchart of the participating subjects.

This was an open-label, randomized, prospective trial that was performed during Ramadan of 1438 (27 May-24 June 2017). A total of 96 patients were recruited from Endocrinology clinic services at Hamad General Hospital, Doha, Qatar. Patients were enrolled within four weeks prior to Ramadan and screened on the week prior to Ramadan. All patients were randomly assigned to two groups. In group A (n= 50) patients took levothyroxine 30 minutes before breaking the fast at sunset (iftar), and in group B (n= 46) patients took it 30 minutes before an early morning meal before sunrise (suhour) [6]. The study was approved by the institutional review board of Hamad Medical Corporation (IRB#15138). The flowchart of the participating subjects is presented in Figure 1.

### 2.2. Inclusion Criteria

We included both genders (male and female) patients with primary hypothyroidism who had stable TSH over the last 6 months prior to the study period aged between 18 and 70 years. Moreover, we enrolled patients who planned to fast during Ramadan.

### 2.3. Exclusion Criteria

Patients with any end organ damage, pregnancy, thyroid cancer, or patients not adhering to medications were excluded. We also excluded patients who had a clinical contraindication to observe fasting.

### 2.4. Data Collection

The anthropometric parameters were assessed 2 weeks before and two weeks after Ramadan. We measured body weight (kg) and subsequently calculated BMI at each visit. A significant change in body weight might affect the level of TSH as levothyroxine dose is related to body weight [4]. Blood samples for TSH and free T4 were obtained at each visit. Information on the duration of hypothyroidism and details on coexisting comorbidities were also obtained. During the second visit we collected individual information on total number of days fasted during the holy month. A questionnaire about compliance and convenience of the timing of levothyroxine intake during the Ramadan fasting was given to all patients [6].

### 2.5. Sampling Method

For a statistically significant difference between the two groups a clinical cut-point for TSH of 1 mIU/ml is considered to be clinically significant. With a standard deviation (SD) of 1.5 mIU/ml, alpha=0.05, power=80%, a minimum of 36 patients will be required in each study arm and a total of 72 patients. With an estimated 20% dropout, at least 90 study subjects will be required.

### 2.6. Randomization

We randomized the patients according to Block randomization method into two groups. The subjects were given numbers, all even numbers were subjected to group A, and all odd numbers were subjected to group B.

### 2.7. Primary Outcomes

To evaluate the difference in TSH level between the two treatment groups.

### 2.8. Secondary Outcomes

To determine the patient preference (by clinical interview), body weight, and blood pressure.

### 2.9. Statistical Analysis

The statistical analysis was performed using SPSS version 20.0 (IBM, Armonk, USA).

All data in the tables and text were manifest as standard deviations and means. The thyroid hormone level variables between the levothyroxine dosage in suhour and iftar were measured by performing an independent samples t-test. All P values less than 0.05 were considered to be statistically significant [7].

## 3. Results

In total 96 hypothyroid patients, who observed fasting during Ramadan participated in this study. Adding to the general fact, women are more susceptible to hypothyroidism, the majority (85 out of 96) of these participants, were female patients (88.5%). Among those patients, the majority of them belong to Qatari or Arab nationality, with minority belonging to other nationalities. In addition to hypothyroidism, the majority of the patients were identified to have comorbidities, such as diabetes mellitus type-2, hypertension, dyslipidemia, and obesity. The patients were randomized between the two groups (Groups A and B); demographic variations in each group were balanced, to obtain an unbiased interpretation. Diabetes mellitus type 2 and dyslipidemia were the predominant comorbidities. The demography of the study population (96 patients) was tabulated in Table 1.

Clinical parameters of the patients, i.e., Weight, BMI, and levels of TSH and FT4, were being evaluated in all patients, before and after Ramadan, and were compared within the group and between the groups for significance. The results of clinical parameters in patients of both groups pre-Ramadan and post-Ramadan are being tabulated in Table 2.

A significant increase in average TSH values was observed in both groups after Ramadan, ranging from 1.99 to 3.28 in Group A and ranging from 1.54 to 3.28 in Group B, which shows doubling in TSH values in postfast period comparing the prefast values. There was no significant difference in TSH variations between the two groups after Ramadan. FT4 levels did not show any significant difference between the groups under both pre- and postfast conditions.

**Figure 2:**
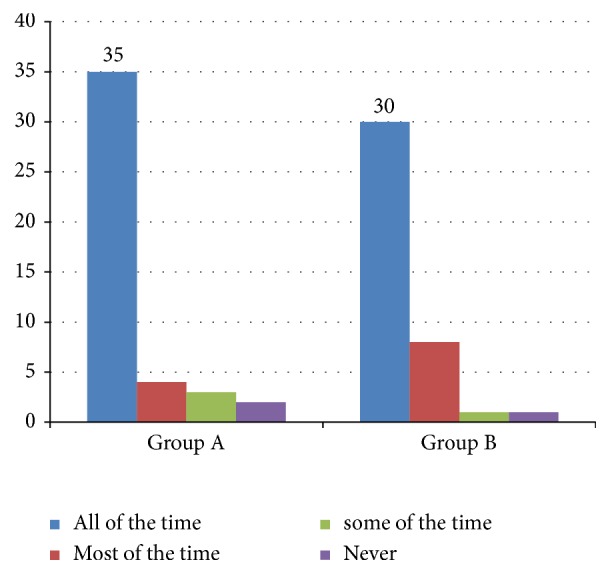
Compliance of the patients enrolled for the study.

**Figure 3:**
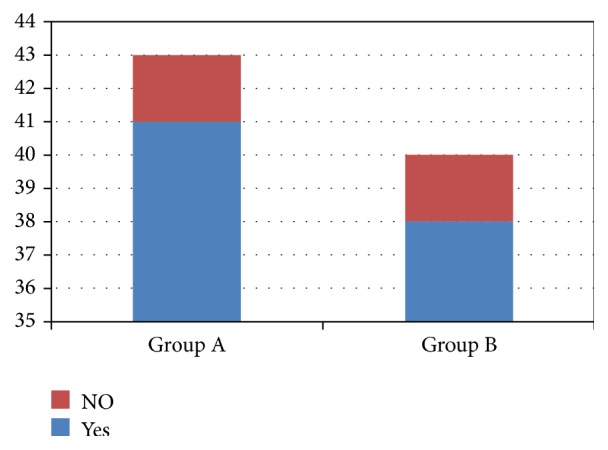
The convenience of the patients to take part in the study.

The majority of patients who took part in the study were present throughout the study, with a small number of patients being irregular and very few discontinued participations halfway through the study. Graphical representation of compliance of the patients in the study is represented in Figure 2. The patients were asked about the convenience of following the treatment (convenience of tablet timing only). The majority of the patients reported that it was convenient to take medications at designated timings. The graphical representation of the patient's convenience is being shown in Figure 3.

## 4. Discussion

This study is one among the few reports that looks into management of hypothyroidism during fasting. Not many studies have reported the significance of the time of administration of levothyroxine during fasting. In our study, TSH increased during Ramadan in both groups, and the increase was statistically significant. There was no significant difference between both groups. Another study also reported a significant increase in TSH throughout Ramadan fasting, but the mean levels showed a steady normal limit before and after Ramadan [8]. Another study on Ramadan fasting and hypothyroidism suggest an increase of 25-50 mcg daily dose of levothyroxine from the start of Ramadan fasting and continuing it till 15-20 days of post-Ramadan [9].

Till date, very few studies have been done specifically looking at TSH variations based on change of timing of administration of levothyroxine during Ramadan [7, 10, 11]. Some studies have suggested that proper administration of levothyroxine during Ramadan is an hour before suhour; however, this suggestion was not followed by most of the patients, as they feel it was difficult to wake up early in the morning [12]. Studies suggest that levothyroxine absorption is up to 80% when it is administered 60 minutes prior to meals, and absorption is decreased up to 60% when administered after meals [12]. This variation in absorption of levothyroxine contributes to altering the TSH levels. However, many other studies did not report any significant change in dose requirement, TSH levels, or variation in the quality of life in morning and evening dose of levothyroxine during Ramadan and/or normal days [13].

Grapefruit, coffee, walnuts, soybeans, dried prunes, orlistat, bisphosphonates, calcium carbonate, antacids, proton pump inhibitors, carbamazepine, sevelamer, rifampicin, and phenytoin are some of known foods and drugs that impair levothyroxine absorption [14]. Interference with levothyroxine absorption has shown to be adversely affected with fiber-enriched diet, soya, charcoal, coffee, raloxifene, iron sulfate, sucralfate, cholestyramine resin, and aluminum antacids [15]. In this study we did not take a diet diary and did not look for medications that might interfere with levothyroxine absorption. This is one of the limitations of our study. The bedtime dosing of levothyroxine ingestion is recommended to be at least two hours after dinner, to avoid interference with food and levothyroxine absorption [13].

Since the adsorption of levothyroxine itself remains a question, further research is to be performed on increasing the levothyroxine dosage by 10-20%, as this could increase the amount of levothyroxine absorbed into the blood and thereby could lead to better management of TSH levels.

Since there are not many previous reports in this field of study, further research and observations are needed to identify the appropriate time of administration of levothyroxine and also the appropriate dose of levothyroxine during the fasting period, to manage hypothyroidism.

Our study showed a significant increase in TSH in both groups, although mean TSH in both groups remained in the normal range. We attribute that to several reasons. In group A, although most of the patients said that they were compliant with the instructions given to them to wait 30 minutes before eating their meal, we think that most of the patients did not wait as long as recommended or might have ingested food or drugs that interfere with absorption. Taking levothyroxine 30 min before iftar is challenging since patients may be unable to wait for this period after about 15 hours of fast. As for group B, the ingestion of fatty meals in Ramadan might delay stomach emptying; thus 2-3 hours after food might not be a sufficient time to wait after a meal in order to take the levothyroxine dose. Compliance in Group B might have also contributed to the rise in TSH. A study done in Ramadan 2012 that examined the effect of giving the levothyroxine tablet at bedtime concluded that nearly 75% patients could not manage an interval of 2 hours between dinner and drug [16]. Increasing levothyroxine dose by 25% might be an option for future research. Data from published research on hypothyroidism and Ramadan fasting suggest increasing the dose of LT4 by 25-50 mcg daily from beginning of Ramadan and continuing the increased dose until 15-20 days after Ramadan [8]. A study conducted on Ramadan fasting among normal subjects showed a significant gradual rise in TSH throughout the fasting month (pre-Ramadan TSH 3.34±0.337; near end of Ramadan TSH 4.61 ± 0.375), though the mean levels remained within normal limits and pre-Ramadan levels were reattained well after the end of Ramadan (TSH 5 months after Ramadan 3.48 ± 0.176) [9]. Our study results are in concordance with the above-mentioned studies.

The strengths of our study include (1) having a relatively large number of patients participating in the study and completing both visits; (2) randomization that might have contributed to decrease in bias; (3) blood samples which were collected within 2 weeks before and after Ramadan; thus we can contribute any TSH change to fasting Ramadan.

The main limitation of our study is that there was no diet diary and no collection of data on the ingestion of food/medication that might interfere with levothyroxine absorption.

Perfect timing for taking levothyroxine tablet remains a problem to be solved. Until then, patients might be given two options for taking it at iftar time or before suhour. The choice depends on the lifestyle of each patient and the convenience of timing. We should emphasize the importance of taking the tablet on an empty stomach and advice on the effect of food on levothyroxine absorption.

## 5. Conclusion

Observations of this current study do not provide a strong recommendation on the ideal time of administration of levothyroxine during Ramadan fasting to manage hypothyroidism. TSH levels were significantly increased in both patient groups who took levothyroxine at iftar time and those who took it at suhour time.

Further studies and observations are required to assess the ideal timing and optimal dose alterations of levothyroxine during the fasting month, to improve the management of hypothyroidism.

## Figures and Tables

**Table 1:** Demography of the study population.

	Group A	Group B	P value
Age, Mean (SD), years	45.1 (12.5)	45.1 (12.7)	0.984
Gender, No. (%)			0.312
Female	43 (86%)	42 (91.3%)	
Male	7 (14%)	4 (8.69%)	
Nationality, No. (%)			0.001
Qatari	31 (62%)	21 (45.7%)	
Arabs	15 (30%)	10 (21.7%)	
Others	4 (8%)	15 (32.6%)	
Days Fasted, Mean (SD)	25.9(4.1)	25.4 (2.9)	
Duration of illness, Years (SD)	6.4 (4.1)	7.9 (5.3)	0.138
Comorbidities	42 (84%)	34 (73.9%)	0.168
DM2	19 (38%)	16 (34.8%)	0.451
Obesity	10 (20%)	10 (21.7%)	0.512
HTN	11 (22%)	6 (13%)	0.193
Dyslipidemia	16 (32%)	18 (39.1%)	0.303

**Table 2:** Changes in clinical data between Group A and Group B.

	Group A			Group B			P value^∧^
	Pre-Ramadan	Post-Ramadan	P-value	Pre-Ramadan	Post-Ramadan	P-value	
Weight, Mean (SD), Kg	81.4(15.5)	83 (20.2)	0.423	79.9(16.7)	79.4(16.8)	0.117	0.245
BMI, Mean (SD)	31.8 (6)	31.9 (6.1)	0.338	31.4 (5.8)	31.3 (6.1)	0.332	0.346
TSH, Mean (SD), mIU/l	1.99 (1.2) *∗*	3.28 (2.6) *∗*	0.001	1.54 (1.1) *∗*	3.28 (4.8)*∗*	0.009	0.357
FT4, Mean (SD), pmol/l	14.2 (2.2) *∗*	13.4(2) *∗*	0.017	14.7 (2.1) *∗*	13.6 (2.3)*∗*	0.001	0.746

(i) *∗*p<0.05, level of significance within each group.

(ii) ^∧^Level of significance between the two groups.

## Data Availability

Data are available from Wajiha Abu Haliqa, primary investigator, email: WHALIQA@hamad.qa, for researchers who meet the criteria for access to confidential data. The data used to support the findings of this study including data collection sheet and statistical analysis are restricted by the Hamad Medical Corporation Ethical Board in order to protect patient privacy.
